# Skeletal Muscle Involvement in Friedreich Ataxia

**DOI:** 10.3390/ijms25189915

**Published:** 2024-09-13

**Authors:** Elisabetta Indelicato, Julia Wanschitz, Wolfgang Löscher, Sylvia Boesch

**Affiliations:** 1 Center for Rare Movement Disorders Innsbruck, Department of Neurology, Medical University of Innsbruck, 6020 Innsbruck, Austria; sylvia.boesch@i-med.ac.at; 2 Unit for Neuromuscular Disorders and Clinical Neurophysiology, Department of Neurology, Medical University of Innsbruck, 6020 Innsbruck, Austria

**Keywords:** Friedreich Ataxia, skeletal muscle, frataxin, mitochondria, transcriptomics, proteomics, biomarker

## Abstract

Friedreich Ataxia (FRDA) is an inherited neuromuscular disorder triggered by a deficit of the mitochondrial protein frataxin. At a cellular level, frataxin deficiency results in insufficient iron–sulfur cluster biosynthesis and impaired mitochondrial function and adenosine triphosphate production. The main clinical manifestation is a progressive balance and coordination disorder which depends on the involvement of peripheral and central sensory pathways as well as of the cerebellum. Besides the neurological involvement, FRDA affects also the striated muscles. The most prominent manifestation is a hypertrophic cardiomyopathy, which also represents the major determinant of premature mortality. Moreover, FRDA displays skeletal muscle involvement, which contributes to the weakness and marked fatigue evident throughout the course of the disease. Herein, we review skeletal muscle findings in FRDA generated by functional imaging, histology, as well as multiomics techniques in both disease models and in patients. Altogether, these findings corroborate a disease phenotype in skeletal muscle and support the notion of progressive mitochondrial damage as a driver of disease progression in FRDA. Furthermore, we highlight the relevance of skeletal muscle investigations in the development of biomarkers for early-phase trials and future therapeutic strategies in FRDA.

## 1. Introduction

Friedreich Ataxia (FRDA) is an autosomal recessive neuromuscular disorder caused by a deficiency of the mitochondrial protein frataxin [1]. In the majority of cases, the associated molecular defect is a biallelic GAA-repeat expansion in the first intron of the frataxin gene (*FXN*) [1]. Approximately 4% of patients are compound heterozygote for an intronic GAA-expansion and one canonical *FXN* variant (point mutation, deletion) [1]. Pathologically expanded GAA-stretches hinder *FXN* transcription by interfering with mRNA elongation and, most of all, via induction of repressive chromatin changes [2,3,4,5]. Cumulative evidence underpins a role for frataxin as an accelerator of a key enzymatic step in the synthesis of iron–sulfur clusters (ISCs) [6], the prosthetic groups of several respiratory chain enzymes and aconitase. A plethora of biochemical and functional studies clearly linked frataxin deficiency with impaired ISC synthesis [5,6,7,8,9] and reduced mitochondrial function and adenosine triphosphate (ATP) production [10,11,12,13,14,15].

FRDA is the most common early-onset inherited ataxia in the Caucasian population [16]. The most prominent symptom is a progressive balance and coordination disorder resulting from an affection of dorsal root ganglia, dorsal column of the spinal cord, peripheral sensory nerves, and, to a lesser extent, cerebellar involvement [17]. The length of expanded GAA repeats, and especially of the shorter one (GAA1), shows a strong inverse correlation with the age of onset of ataxia and the severity of the phenotype [18,19,20,21]. Longer expansions are thus associated with early-onset disease and faster disease progression [20,22].

Beyond the neurological syndrome, FRDA also displays a muscle phenotype, affecting both the heart and the skeletal muscle. Typical FRDA cardiomyopathy presents with an usually mild hypertrophy of the left ventricle with preserved ejection function and no obstruction [23]. Its occurrence is related to the underlying genetic severity [24]. Up to 75% of individuals with early-onset disease (<25 years of age) display signs of cardiomyopathy [25], while heart involvement is far less frequent when the disease manifests later. Supraventricular arrhythmias and left ventricle remodeling with ejection fraction decline may develop during the disease course [26,27]. Although ataxia causes the highest disease burden, cardiomyopathy is the main determinant of reduced survival [27,28,29].

While, for obvious reasons, most of the attention in FRDA has been paid to the involvement of cardiac muscle, frataxin deficiency does not spare the skeletal muscle. Skeletal muscle accounts for up to 50% of the total body mass [30,31]. *FXN* mRNA and protein expression in skeletal and heart muscle is comparable and among the highest in the human body [32,33]. Functional imaging, histology and multi-omics techniques in both disease models and patients revealed a skeletal muscle disease phenotype in FRDA [10,11,34,35,36]. Clinically, evidence of skeletal muscle involvement can be highly variable and appears to be more pronounced in the setting of more severe phenotypes (longer GAA repeats, compound heterozygous with null *FXN* variants) [33,37,38,39]. In a study of children with FRDA, muscle weakness was more pronounced in the proximal leg and correlated with increased ultrasound muscle density in the absence of leg atrophy, a constellation of findings suggestive of myopathic involvement [38]. Skeletal muscle involvement contributes to the marked fatigue and reduced exercise capacity that outlasts the degree of neurological impairment or the expected effect of secondary deconditioning due to reduced mobility [39]. Muscle weakness also contributes to the restrictive impairment of respiratory function [40], which is associated with higher surgical morbidity in FRDA patients [41].

In this review, we provide a novel overview on the existing knowledge on skeletal muscle in FRDA. We emphasize the importance of skeletal muscle research in translational studies, particularly in developing biomarkers for early-phase clinical trials and formulating future therapeutic strategies.

## 2. Skeletal Muscle Involvement in FRDA: Findings from Histology and Functional Imaging

In mitochondrial myopathies, muscle biopsy is a cardinal investigation [42]. Cytochrome c oxidase (COX) histochemistry is central to the assessment [43]. It reflects the activity of complex IV of the respiratory chain, three subunits of which are encoded by mitochondrial DNA (mtDNA). Patients with mtDNA mutations have a mosaic of ‘COX-negative’ fibers, reflecting heteroplasmy, i.e., differences in the amount of mutated mtDNA between myofibers [43]. COX-negative fibers are best detected by serial staining for COX followed by complex II (succinate dehydrogenase) staining [43]. Succinate dehydrogenase is encoded entirely by nuclear genes and therefore maintains normal activity irrespective of heteroplasmy levels. Myofibers may also show compensatory mitochondrial proliferation. This can be seen as a ‘ragged’ appearance in fibers stained blue by succinate dehydrogenase or red by Gomori trichrome staining [44]. Additional to histochemical clues, or when these are not evident, respiratory chain enzyme analysis can help identify mitochondrial abnormalities [42].

Reports on histological findings in skeletal muscle in FRDA are sparse (see Table 1). FRDA patients usually do not present the prominent histological and biochemical alterations classically associated with mitochondrial myopathy. Cytochrome-c oxidase-negative fibers and reduced activity of the respiratory chain enzymes may be found, more consistently for complex I [36,37]. An anecdotal report described overt histological features of a mitochondrial myopathy in a child with FRDA, including the presence of ragged red fibers and ragged blue fibers [37].

In a small cohort study [36], morphological and fiber type analysis of gastrocnemius muscle biopsies revealed only mild myopathic changes, including selective type II fiber atrophy. Fiber-type grouping, a finding suggestive of muscle denervation, was described in both this work and in an earlier case report [45].

Few studies have used magnetic resonance (MR)-based functional imaging techniques in skeletal muscle [11,35,46]. ATP-generating capacity has been shown to be lower in the skeletal muscle of individuals with FRDA by using ^31^P MR spectroscopy [11]. This imaging technique demonstrates in the calf muscle of FRDA patients a more rapid depletion of phosphocreatine, the readily available source for ATP generation during maximal muscular effort, compared to healthy controls [11,35]. Furthermore, the regeneration of phosphocreatine after maximal exhaustive exercise is delayed in the calf muscle of individuals with FRDA [35,47]. By means of “chemical exchange saturation transfer” (CEST) MR imaging, the opposite phenomenon can be studied, i.e., the decline in free creatine after exercise, as phosphocreatine is regenerated. The CEST technique has a superior spatial resolution to ^31^P MR spectroscopy, enabling a muscle group-specific assessment [48]. In FRDA patients, CEST-based MR identified a prolonged decline in free creatine only in the gastrocnemius muscle group, as opposed to the soleus [46]. This specific pattern of involvement highlighted by CEST MR may be related to the different myofiber content in the two muscles. Indeed, gastrocnemius muscle has a higher density of fast twitch, glycolytic myofibers, which have greater phosphocreatine content at rest [49]. Consistent with the findings described above, near infrared muscle spectroscopy (NIRS) also shows a prolonged recovery from exercise-induced deoxygenation in the calf muscle of FRDA [50]. A summary of the reviewed studies is provided in Table 1.

**Table 1:** Summary of studies addressing skeletal muscle involvement in FRDA patients.

Study	Methodology	Findings
**Histology and immunohistochemistry**		
Gallagher 2002 [37]	Histology	Prominent mitochondrial myopathy in one patient with skeletal muscle biopsy showing ragged red fibers and ragged blue fibers
Nachbauer 2012 [36]	Histology, immunohistochemistry, enzymatic assays	Mild myopathic features, single cytochrome c oxidase-negative fibers, reduction in complex I activity
**Imaging studies**		
Lodi 1999 [11]	^31^P MR spectroscopy	Reduced ATP synthesis rate in calf muscle of FRDA patients.
Vorgerd 2000 [47]	^31^P MR spectroscopy	Prolonged recovery of phosphocreatine after exercise in calf muscle of FRDA patients.
Lynch 2002 [50]	Near infrared spectroscopy	Prolonged recovery from exercise-induced deoxygenation in calf muscle of FRDA patients.
Nachbauer 2013 [13]	^31^P MR spectroscopy	Prolonged recovery of phosphocreatine after exercise in calf muscle of FRDA patients.
Schur 2022 [46]	CEST MR imaging	Prolonged decline in free creatine after exercise in gastrocnemius muscle of FRDA patients.
Sival 2011 [38]	Ultrasound imaging	Increased muscle ultrasound density in the biceps, quadriceps, and tibialis anterior muscles.
**Exercise capacity testing**		
Drinkard 2010 [39]	Incremental exercise testing	Significantly impaired exercise capacity in children and adolescents with FRDA
**Omics studies**		
Indelicato 2023a [15]	RNA-Sequencing	1873 differentially expressed genes in gastrocnemius muscle biopsy of FRDA patients, with mostly mitochondrial terms, but also, i.e., lipid metabolism, including the hormone leptin.
Indelicato 2023b [34]	Mass spectrometry	228 differentially expressed proteins in gastrocnemius muscle biopsy of FRDA patients, 74% of which are target of *NRF2*.

## 3. Skeletal Muscle and Regulation of Metabolism in FRDA: Insights from Disease Models

Skeletal muscle involvement was addressed in a number of studies in murine models of FRDA, which particularly highlighted alterations in muscle-related metabolic processes in the setting of frataxin deficiency [51,52,53,54]. Indeed, as highly active tissue, skeletal muscle plays a pivotal role in the regulation of carbohydrate and lipid metabolism [55,56]. An early microarray study focused on skeletal muscle involvement in the KIKO transgenic mouse model of FRDA. KIKO mice are compound heterozygous for a null *FXN* allele and an *FXN* allele carrying a short GAA expansion; they have a 25–36% residual frataxin protein expression compared to wild type mice and display a very mild phenotype [57]. KIKO mice showed multiple differences in gene expression in skeletal muscle when compared to control littermates. Of these, lipid metabolism was identified as the top differentially expressed pathway in skeletal muscle [53]. Particularly, a number of gene encoding enzymes involved in the metabolism of fatty acids (*SCD5*, *ACLY*, *ACSS2*, *AACS*) were upregulated. Numerous alterations in lipid metabolism-related processes were observed in both the skeletal muscle and the liver of KIKO mice. The downregulation of several contractile proteins expressed in slow-twitch fibers in the skeletal muscle of KIKO mice suggested a shift towards fast, more anaerobic, less oxidative fiber types. Notably, transcriptional changes observed in skeletal muscle were significantly more pronounced than those observed in cardiac muscle of KIKO mice (n = 321 versus n = 174 differentially expressed genes, respectively) [53]. In general, the transcriptional alterations observed in the skeletal muscle, heart, and liver of KIKO mice were consistent with a disruption in the peroxisome proliferator-activated receptor γ (*PPARγ*)/peroxisome proliferator-activated receptor gamma coactivator-1α (PGC-1α)-related pathway. *PPARγ* and its transcriptional coactivator PGC-1α regulate mitochondrial biogenesis, carbohydrate and lipid metabolism, and the remodeling of muscle tissue to a fiber-type composition that is metabolically more oxidative and less glycolytic in nature [58,59,60,61]. These early findings in KIKO mice provided a rationale for further investigations on the contribution of the *PPARγ*/PGC-1α pathway in FRDA. Recently, leriglitazone, a *PPARγ* agonist, entered clinical trials with a first phase 2 study completed (ClinicalTrials.gov: NCT03917225; EudraCT: 2018-004405-64) [62].

Similarly to the brain and the heart, skeletal muscle is capable of utilizing ketone bodies as an alternative energy source during periods of fasting or low carbohydrate availability [63,64]. The ketone bodies produced by the liver are converted in target extrahepatic tissues to acetoacetyl-CoA, which is then fueled into the Krebs cycle for the production of ATP [63]. The key rate-limiting enzyme in this process, 3-oxoacid CoA transferase 1 (OXCT1), has been shown to interact with frataxin in a study using immunoprecipitation with anti-frataxin antibodies [51]. The same study showed a correlation between changes in frataxin expression and OXCT1 expression in different cell types. This interaction was further investigated in KIKO mice [51]. OXCT1 was reduced in skeletal muscle from both KIKO mice and FRDA patient samples. In KIKO mice, plasma ketone body levels were significantly elevated compared to control mice. At the same time, mass spectrometry analysis revealed reduced acetyl-CoA levels in skeletal muscle homogenates from KIKO mice as compared to controls upon fasting. These results suggest that a deficit in ketone body utilization occurs in skeletal muscle of KIKO mice. Based on these findings, the authors hypothesized that a reduction in skeletal muscle OXCT1 in FRDA may contribute to prolonged post-exercise recovery and exercise intolerance. Prolonged exercise stimulates the ability of skeletal muscle to take up ketones from the blood [55,63,64] and increases OXCT1 activity in mice [65,66]. This may explain the observation that long-term exercise training prevents the development of exercise intolerance in KIKO mice [52]. Translating these findings in human studies, impaired ketone body utilization in FRDA could similarly lead to inefficient ATP production and energy deficits, particularly under conditions of exercise or fasting stress, as seen in clinical settings [39].

Children and adults with FRDA have lower lean mass in the arms and legs [54]. Distinguishing between a primary effect of frataxin loss and consequences of deconditioning is challenging. Experiments in an inducible frataxin depletion mouse model (FRDAkd) [67] showed reduced muscle mass gain and smaller myofibers with increasing frataxin depletion [54]. At the molecular level, this phenotype was accompanied by decreased global protein translation and activation of the “integrated stress response”, an intracellular signaling network that reprograms gene expression in response to a variety of conditions to maintain protein homeostasis [54]. A summary of the studies addressing skeletal muscle involvement in FRDA murine models is provided in Table 2.

## 4. Omics Studies in Skeletal Muscle from FRDA Patients

The advances of omics techniques provided unique insight into the molecular biology and pathophysiology underlying a number of physiological and disease conditions. Based on RNA-sequencing data of the entire human transcriptome, which includes 20,162 protein-coding genes, 64% of these genes are expressed in skeletal muscle, and 921 genes show higher expression in skeletal muscle compared to other tissues [32,68]. As expected, genes involved in contraction, actin filament-based processes, muscle organ development, and muscle filament sliding are particularly over-represented in skeletal muscle. Moreover, the cardiac muscle and skeletal muscle have by far the highest fraction of transcripts encoding mitochondrial proteins of all analyzed tissues, corresponding to 28–32% of the total mRNA pool [68]. Interestingly, a number of genes are simultaneously enriched in both cardiac and skeletal muscles, adipose tissue, and liver, mainly represented by genes implicated in metabolic processes and enzymatic activities. Transcriptomics studies revealed in particular a high correlation in the expression of protein coding genes between both skeletal and cardiac muscle and the adipose tissue [68].

**Figure 1:**
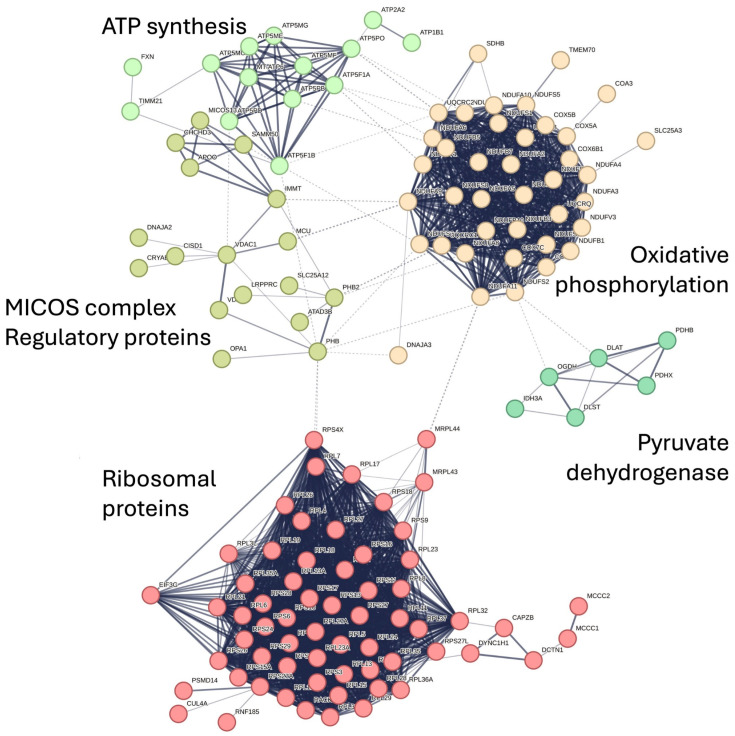
Interactome of differentially expressed proteins in skeletal muscle of FRDA adapted from the STRING database (reference: Indelicato et al. 2023b, [34]). MICOS: mitochondrial contact site and cristae organizing system.

**Figure 2:**
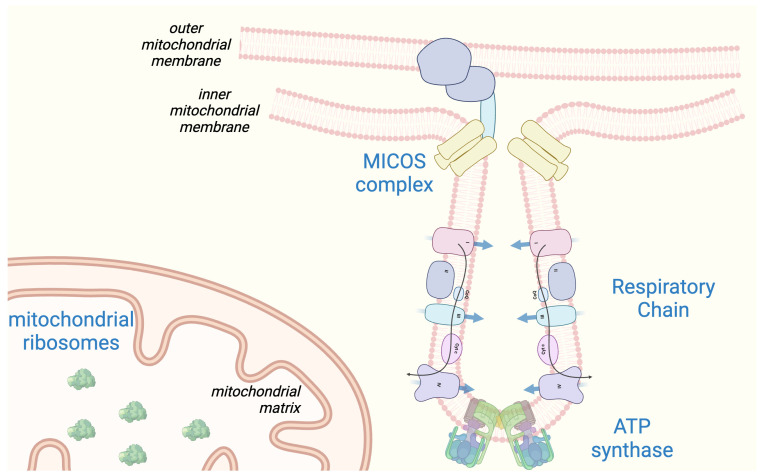
Mitochondrial localization of top downregulated proteins in skeletal muscle in FRDA (reference: Indelicato et al. 2023b, [34]). MICOS: mitochondrial contact site and cristae organizing system. Figure created with BioRender.com.

Recently, our group applied transcriptomic and proteomic analysis in gastrocnemius muscle biopsies from FRDA patients and controls. Overall, 1873 genes were differentially expressed between FRDA and control muscle, with 1144 of them showing a >2-fold change in gene expression. The extent of the gene expression changes in skeletal muscle was far more marked than those detected in non-affected cells, such as peripheral blood mononuclear cells (PBMCs) or fibroblasts from FRDA patients [69,70,71]. More importantly, gene expression changes in skeletal muscle biopsies were far more marked than those found in sensory neurons derived from induced pluripotent stem cells (iPSCs) [72,73]. Pathways related to mitochondrial composition and function dominated the set of downregulated genes in skeletal muscle [15]. These included not only terms related to oxidative phosphorylation but also ISC assembly, Krebs cycle, mitochondrial transport proteins, mitochondrial structural/regulatory proteins, and a large group to mitochondrial ribosomes. *FXN* mRNA was about ~3.5-fold decreased in FRDA samples comparing to controls [15]. Several genes related to lipid metabolism were also listed among the differentially expressed ones, including the hormone leptin, which, with a 4-fold increase, was among the most strongly upregulated mRNA in FRDA skeletal muscle [15]. Circulating leptin derived from adipocytes has been associated with a dysmetabolic phenotype, obesity, and inflammation [74]. Paracrine-secreted leptin may instead bear a protective effect against mitochondrial dysfunction and support energetic homeostasis [75]. A similar upregulation of leptin was found in FRDA patient-derived lymphocytes and in the skeletal muscle of frataxin-deficient mice [69]. When changes at the proteome level were analyzed, mass spectrometry identified 228 differentially expressed proteins, of which 227 were downregulated (see also Figure 1) [34]. Frataxin protein levels were ~1.5-fold decreased in FRDA samples compared to controls. The two largest cluster consist of ribosomal proteins and structural components from all the complexes of the oxidative phosphorylation. These include not only proteins containing ISCs but also several subunits of complex V/ ATP synthase. Notably, ATP synthase plays not only a pivotal role in energy production, but also serves a structural function. Indeed, the assembly of dimeric ATP synthase complexes contributes to the shaping of the cristae (see Figure 2). Inherited disorders with reduced levels of ATP synthase result in abnormalities of mitochondrial morphology [76]. To this concern, an additional mitochondrial protein cluster that is downregulated in FRDA comprises proteins of the “mitochondrial contact site and cristae organizing system” (MICOS) complex, a large protein apparatus located at the inner mitochondrial membrane that also plays a role in shaping the morphology of cristae (see Figure 2). [77]. Almost 75% of downregulated proteins in calf biopsies are the target of the transcription factor NRF2, a master regulator of mitochondrial homeostasis which shows a defective activation in FRDA. This is particularly noteworthy, as the first treatment approved for the treatment of FRDA, omaveloxolone, acts as activator of NRF2 [78]. Omaveloxolone has been approved based on the results of the MOXIe trial [79], which showed a significant improvement in the neurological scores in the treatment arm as compared to placebo over a period of one year. NRF2 is also involved in defense against oxidative stress, whose signature has been found in several FRDA models and in patient-derived cells [12,52,80,81,82]. These findings set the rationale for the early investigation of antioxidant compounds in the treatment of FRDA [83]. Remarkably, the only upregulated protein in calf muscle of FRDA was the glutathione S-transferase mu 4 (GSTM4), an enzyme involved in detoxification of electrophilic compounds and oxidative stress [34]. This finding is consistent with increased oxidative stress in skeletal muscle as well, which in turn may promote mitochondrial damage in a vicious cycle. Mitochondria are also involved in Ca^2+^ homeostasis, which is critical for contraction in both cardiac and skeletal muscle. Impaired mitochondrial Ca^2+^ uptake has been shown in *FXN* knockdown cardiomyocytes, which was restored by treatment with antioxidants [84].

Skeletal muscle represents a primary site for glucose disposal within the human body and is involved in the pathophysiology of insulin resistance in diabetes mellitus type 2 [56]. In physiological conditions, skeletal muscle overexpresses a large number of proteins related to different metabolic processes [68]. Several of these proteins were downregulated in FRDA skeletal muscle as compared to control samples. *MCCC1* and *MCCC2* code for the two subunits of 3-methylcrotonyl-CoA carboxylase (MCC), a heterodimeric biotin-dependent enzyme that catalyzes the fourth step in the leucine catabolic pathway [85]. Leucine is a nutritionally essential branched-chain amino acid and increases protein synthesis through activation of the mammalian target of rapamycin (mTOR) signaling pathway in skeletal muscle, adipose tissue, and placental cells [86,87]. Among all the essential amino acids, leucine represents the most potent stimulator of mTOR (~3-fold increase in mTOR phosphorylation activity compared to other essential amino acids) [86,87]. The cluster of downregulated proteins in FRDA skeletal muscle also included glycogen synthase 1 (GYS1), which is responsible for the addition of glucose units to glycogen, and the protein kinase AMP-activated non-catalytic subunit beta 2 (PRKAB2), a regulatory subunit of the AMP-activated protein kinase (AMPK). AMPK is an energy-sensing enzyme that monitors cellular energy status. In response to cellular metabolic stressors, AMPK is activated and, through phosphorylation, exerts control over the activity of key enzymes involved in regulating de novo biosynthesis of fatty acids and cholesterol [88].

## 5. Skeletal Muscle as Disease State Readout for Interventional Studies

A significant gap in FRDA research is the absence of an easily accessible biomarker derived from biofluids to monitor disease progression, similar to how neurofilaments are used in motoneuron disease [89]. A number of therapeutic approaches under investigation in FRDA aim at increasing frataxin level, including gene therapy, epigenetic therapy, and direct frataxin addition (reviewed in [90]). Frataxin measurements have traditionally been carried out in PBMCs [91,92] or epithelial cells from buccal swabs [93]. Changes in frataxin levels in these cells may not reflect concordant variations in affected tissues. Although more invasive than a blood withdrawal, skeletal muscle can easily and repeatedly be sampled via punch biopsy, a minimally invasive procedure. The evaluation of alterations in skeletal muscle parameters in response to pharmacological interventions represents a promising avenue for the identification of surrogate endpoints in early-phase clinical trials. This concept has already been explored in the first study on skeletal muscle histology in FRDA, which examined changes in frataxin muscle content and muscle histology before and after an eight-week treatment with recombinant human erythropoietin [36]. In accordance with its function as a potent growth factor, erythropoietin treatment resulted in an increase in the number of mature capillaries in skeletal muscle. No additional alterations were identified in vascular and myogenic markers at the histological level, nor in functional imaging via ^31^P MR spectroscopy following erythropoietin treatment [35,36]. However, subsequent analysis via RNA-sequencing in the same muscle samples revealed that erythropoietin resulted in the upregulation of 37 genes, several of which were involved in the organization of the extracellular matrix, mesenchymal differentiation, cellular migration, and adhesion [15]. Treatment with erythropoietin led to a further marked upregulation of leptin, with ca. 16-fold higher levels in FRDA samples after treatment as compared with controls.

More recently, the change in frataxin levels in skeletal muscle biopsies was selected as the primary endpoint in a phase 1, multiple-ascending dose study investigating the compound DT-216 for the treatment of FRDA. DT-216 (Design Therapeutics, Carlsbad, CA, USA) is a GeneTAC™ gene-targeted chimera small molecule that has been designed to bind specifically to the *FXN* GAA-repeat expansion and modulate the cell’s native transcriptional machinery. DT-216 has been demonstrated to increase frataxin levels in skeletal muscle biopsies from individuals with FRDA [94].

## 6. Discussion

Skeletal muscle represents one of the largest organs in the human body, accounting for up to 50% of the total body mass [30,31]. The primary function of skeletal muscle is to facilitate body movement, maintain posture, and ensure stability through coordinated contraction of multiple muscle groups [30]. Moreover, skeletal muscle plays a role in regulating body temperature by producing heat during contraction, regulating blood flow, and, most importantly, serving as a key regulator of metabolism [30,56]. The expression of *FXN* mRNA in skeletal muscle and cardiac muscle is comparable, with levels among the highest observed in the body [32]. Cardiac involvement in FRDA is clinically more prominent than skeletal muscle involvement. Conversely, the changes in gene expression associated with frataxin deficiency are more pronounced in skeletal muscle than in cardiac muscle in KIKO mice [53]. Beyond tissue-specific responses to frataxin loss, this apparent discrepancy may simply be related to the different workload of cardiac and skeletal muscle. While the first is, of course, continuously active, skeletal muscle is subject to voluntary control and does not undergo obligatory uninterrupted activation. Skeletal muscle may thus better compensate for mitochondrial dysregulation in the setting of frataxin deficiency than myocardium. Despite the less prominent clinical involvement, skeletal muscle in FRDA clearly displays the biochemical and molecular features of frataxin loss [15,34,53]. This is of relevance given that skeletal muscle can be sampled routinely, in contrast to cardiac muscle and the nervous system, which are not as readily accessible. For these reasons, skeletal muscle endpoints were identified as a potential disease state readout at an early stage of drug development for FRDA [35,36,39]. Easily accessible biomarkers capable of capturing target engagement, before a clinical benefit becomes manifest, are highly desirable for early-phase trials in slowly progressing neurodegenerative conditions and are still an unmet need in FRDA. Recent findings from RNA-sequencing reveal that the skeletal muscle transcriptome is sensitive to changes upon pharmacological intervention [15]. A treatment with erythropoietin as short as eight weeks led to significant changes in gene expression in calf muscle biopsies from FRDA patients, which (1) are consistent with the properties of this hormone and (2) are clearly detectable despite skeletal muscle not being its primary target [15]. Assessment of various therapeutic strategies aiming at increasing frataxin, from gene therapy to frataxin protein replacement administered systemically, could benefit from frataxin measurements in skeletal muscle [94].

Calf biopsies from FRDA patients with long-standing disease showed extensive changes in mitochondrial gene and protein expression. Such alterations were far milder, if even present, in experiments performed in iPSC-derived neurons from FRDA patients, a disease model that reflects a rather early disease stage [72]. These findings collectively indicate that a progressive accumulation of mitochondrial damage occurs over the course of the disease, as already hypothesized earlier [14]. The cumulative mitochondrial damage may, in turn, represent a primary driver of disease progression [95,96]. This notion supports the ongoing investigation into therapeutic strategies aimed at improving mitochondrial function in FRDA [97], exemplified by the recent approval of omaveloxolone, a drug targeting mitochondrial dysfunction in FRDA [78].

The primary objective in clinical research in FRDA is the development of a therapy able to treat the neurological and cardiac diseases. Additional strategies are under development to treat specific manifestations of FRDA, such as optic neuropathy [98]. Skeletal muscle is not a primary treatment target in FRDA. Nevertheless, ameliorating the consequence of frataxin loss in skeletal muscle may have a substantial benefit in FRDA individuals by compensating for the mobility deficit via improved physical performance and endurance.

## 7. Conclusions and Future Directions

Skeletal muscle involvement has been poorly addressed in clinical and translational research in FRDA. The advent of gene therapy and protein replacement therapies has reinvigorated the search for biomarkers and disease state readouts for early-phase clinical trials. The strong expression of frataxin in skeletal muscle and the presence of a discernible disease phenotype in this readily accessible tissue provide a compelling rationale for the development of muscle endpoints for clinical trials. Moreover, skeletal muscle represents an ideal tissue for elucidating unclear aspects of mitochondrial dysregulation in FRDA.

## Figures and Tables

**Table 2:** Summary of studies addressing skeletal muscle involvement in FRDA murine models.

Study	Methodology	Findings
Coppola 2009 [53]	Microarrays in gastrocnemius muscle of KIKO mice	321 differentially expressed genes in skeletal muscle (as opposed to n = 174 in cardiac muscle). Downregulation of contractile proteins expressed in slow-twitch fibers. Findings compatible with a dysregulation of the *PPARγ*/PGC-1α pathway.
Zhao 2020 [52]	Western blots and oxygen consumption rate in gastrocnemius muscle of KIKO mice	Long-term voluntary running in KIKO mice improves mitochondrial function and oxidative stress in skeletal muscle without influencing frataxin levels.
Dong 2022 [51]	Enzymatic assays and mass spectrometry in whole skeletal muscle tissue from KIKO mice	Impaired metabolism of ketone bodies in FRDA skeletal muscle via dysregulation of the key enzyme OXCT1, which interacts with frataxin.
Vásquez-Trincado 2022 [54]	Immunoblots and RNA levels in quadriceps and soleus muscle of FRDAkd mice	Reduced muscle mass gain and smaller myofibers with increasing frataxin depletion in mice. Decreased global protein translation and activation of the integrated stress response in skeletal muscle.
